# Supplementary material for: Innate immunity, therapeutic targets and monoclonal antibodies in SARS-CoV-2 infection
Source: PeerJ. 2025 Jun 20;13:e19462. doi: 10.7717/peerj.19462 (PMC12184675; doi:10.7717/peerj.19462)
Supplement: Supplemental Information 2 [file peerj-13-19462-s002.docx]

Editor-in-Chief
PeerJ Journal

**Subject:** Request for Addition of Co-Author to Manuscript (Innate Immunity, Therapeutic Targets and Monoclonal Antibodies in SARS-CoV-2 Infection).

Dear Editor,

I hope this letter finds you well. I am writing to formally request the addition of ***Asim Azhar*** as a co-author to our manuscript titled “Innate Immunity, Therapeutic Targets and Monoclonal Antibodies in SARS-CoV-2 Infection)” (Manuscript ID: **103875**), which is currently under consideration at PeerJ. Azhar has made substantial contributions to the manuscript, warranting authorship based on the journal’s criteria.

***Dr. Azhar*** played a pivotal role in the preparation of Figure 2, titled “**Molecular Mechanisms of SARS-CoV-2-Induced Pulmonary Injury via Apoptotic Pathways**.” He meticulously designed and developed this figure, integrating complex molecular interactions relevant to SARS-CoV-2-induced apoptosis. His expertise significantly enhanced the clarity and scientific rigor of our manuscript’s visual representation of the discussed mechanisms.

In addition, ***Dr. Azhar*** thoroughly edited the entire manuscript, ensuring its compliance with PeerJ’s formatting and editorial requirements. His revisions improved the manuscript’s structure, coherence, and scientific accuracy, addressing critical aspects necessary for a high-quality submission.

Given his substantial intellectual and technical contributions, we kindly request the formal inclusion of ***Asim Azhar*** as a co-author. We believe his addition aligns with the authorship guidelines and accurately reflects his contributions to the work.

We appreciate your consideration of this request and look forward to your positive response. Please let us know if any further details are required to facilitate this update.

Best Regards,
**Dr. Wajihul Hasan Khan, Ph.D**

Virology Unit, Department of Microbiology,

All India Institute of Medical Sciences, Delhi, India, 110029

Mail ID: [wajihulbiotech@gmail.com](mailto:wajihulbiotech@gmail.com)
